# Correction: Mapping the Landscape of Open Access Dashboards - A Dataset for Research and Infrastructure Development

**DOI:** 10.1038/s41597-026-07603-7

**Published:** 2026-06-13

**Authors:** Johannes Schneider, Heinz Pampel

**Affiliations:** 1 Helmholtz-Association, Helmholtz Open Science Office, Telegrafenberg, 14473 Potsdam, Germany; 2 Humboldt-Universität zu Berlin, Berlin School of Library and Information Science (IBI), Dorotheenstr. 26, 10117 Berlin, Germany

Correction to: *Scientific Data* 10.1038/s41597-026-07217-z, published online 29 April 2026

In this Data Descriptor, there were two errors to reference citations in the text.

The text ‘These dashboards enable the communication of the current state of affairs using defined indicators^7^. defines dashboards as follows:…’ should have read ‘These dashboards enable the communication of the current state of affairs using defined indicators. Few^7^ defines dashboards as follows:…’

In addition, the text ‘The use of dashboards to meet information needs in the area of Open Access is gaining increasing importance^14^. published a noteworthy article on Open Access monitoring, which focuses on the landscape of existing dashboards.’ should have read ‘The use of dashboards to meet information needs in the area of Open Access is gaining increasing importance. Salamoura & Tsakonas^14^ published a noteworthy article on Open Access monitoring, which focuses on the landscape of existing dashboards.’

The original Data Descriptor has been updated.

